# *N* ^3^-[(*E*)-Morpholin-4-yl­methyl­idene]-1-phenyl-1*H*-1,2,4-triazole-3,5-diamine monohydrate

**DOI:** 10.1107/S160053681004729X

**Published:** 2010-11-20

**Authors:** V. M. Chernyshev, A. V. Astakhov, V. V. Ivanov, Z. A. Starikova

**Affiliations:** a South-Russia State Technical University, 346428 Novocherkassk, Russian Federation; b A. N. Nesmeyanov Institute of Organoelement Compounds, 119991 Moscow, Russian Federation

## Abstract

In the title compound, C_13_H_16_N_6_O·H_2_O, the mean planes of the benzene and 1,2,4-triazole rings form a dihedral angle of 54.80 (5)°. The N atom of the amino group adopts a trigonal–pyramidal configuration. Conjugation in the amidine N=C—N fragment results in sufficient shortening of the formal single bond. In the crystal, inter­molecular N—H⋯O and O—H⋯N hydrogen bonds link mol­ecules into double layers parallel to the *bc* plane.

## Related literature

The title compound was synthesized according to Astakhov & Chernyshev (2010 ▶). The synthesis of 3,5-diamino-1-phenyl-1,2,4-triazole is described by Steck *et al.* (1958 ▶). Intra­molecular reactions of *N*-substituted amino­methyl­ene malonates accompanied by nucleophilic substitution of malonic ester were described by Sunder & Peet (1980 ▶); Yamazaki *et al.* (1988 ▶); Selic *et al.* (1998 ▶, 2000 ▶); Tkachev *et al.* (2007 ▶). Analogous inter­molecular reaction affording substituted formamidines was described by Rajappa *et al.* (1970 ▶); Bao *et al.* (2008 ▶). For examples of the use of the triazolyl-substituted amidines in the synthesis of annulated heterocycles, see: Dolzhenko *et al.* (2007 ▶, 2008**a* ▶,b* ▶). For crystal structures of substituted 3,5-diamino-1,2,4-triazoles, see: Ried *et al.* (1983 ▶); Dunstan *et al.* (1998 ▶); Chernyshev *et al.* (2006 ▶, 2007 ▶, 2009 ▶). For crystal structures of hetaryl substituted amidines, see: Ryng & Glowiak (1998 ▶); Kurbatov *et al.* (2006 ▶); Xie *et al.* (2007 ▶); Lyakhov *et al.* (2008 ▶); Quiroga *et al.* (2010 ▶). The synthesis of mesoionic [1,2,4]triazolo[4,3-*a*]pyrimidines from *N*-(5-amino-1-*R*-1,2,4-triazol-3-yl)-substituted enamino­esters was described by Chernyshev *et al.* (2010 ▶). For a description of the Cambridge Structural Database, see: Allen (2002 ▶). For values of bond lengths in organic compounds, see: Allen *et al.* (1987 ▶). For the correlation of bond lengths with bond orders between *sp* ^2^ hybridized C and N atoms, see: Burke-Laing & Laing (1976 ▶).
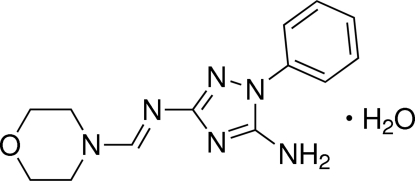


## Experimental

### 

#### Crystal data

C_13_H_16_N_6_O·H_2_O
*M* *_r_* = 290.33
Triclinic, 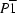

*a* = 8.7886 (7) Å
*b* = 9.0100 (7) Å
*c* = 9.4373 (7) Å
α = 99.938 (1)°
β = 105.933 (1)°
γ = 95.331 (1)°
*V* = 700.00 (9) Å^3^
*Z* = 2
Mo *K*α radiation
μ = 0.10 mm^−1^
*T* = 100 K
0.55 × 0.30 × 0.25 mm

#### Data collection

Bruker APEXII CCD area-detector diffractometer
Absorption correction: multi-scan (*SADABS*; Bruker, 2004 ▶) *T*_min_ = 0.948, *T*_max_ = 0.976
5231 measured reflections
2724 independent reflections
2510 reflections with *I* > 2σ(*I*)
*R*_int_ = 0.015

#### Refinement

*R*[*F* ^2^ > 2σ(*F* ^2^)] = 0.034
*wR*(*F* ^2^) = 0.088
*S* = 1.00
2724 reflections
206 parameters
H atoms treated by a mixture of independent and constrained refinement
Δρ_max_ = 0.18 e Å^−3^
Δρ_min_ = −0.29 e Å^−3^

### 

Data collection: *APEX2* (Bruker, 2004 ▶); cell refinement: *SAINT* (Bruker, 2004 ▶); data reduction: *SAINT*; program(s) used to solve structure: *SHELXS97* (Sheldrick, 2008 ▶); program(s) used to refine structure: *SHELXL97* (Sheldrick, 2008 ▶); molecular graphics: *ORTEP-3* (Farrugia, 1997 ▶); software used to prepare material for publication: *SHELXTL* (Sheldrick, 2008 ▶), *publCIF* (Westrip, 2010 ▶) and *PLATON* (Spek, 2009 ▶).

## Supplementary Material

Crystal structure: contains datablocks I, global. DOI: 10.1107/S160053681004729X/cv2798sup1.cif

Structure factors: contains datablocks I. DOI: 10.1107/S160053681004729X/cv2798Isup2.hkl

Additional supplementary materials:  crystallographic information; 3D view; checkCIF report

## Figures and Tables

**Table 1:** Hydrogen-bond geometry (Å, °)

*D*—H⋯*A*	*D*—H	H⋯*A*	*D*⋯*A*	*D*—H⋯*A*
N5—H5*A*⋯O1^i^	0.89 (2)	2.08 (2)	2.929 (2)	159 (1)
N5—H5*B*⋯O2^ii^	0.89 (2)	2.04 (2)	2.906 (2)	164 (1)
O2—H2*A*⋯N3	0.89 (2)	2.07 (2)	2.929 (2)	164 (1)
O2—H2*B*⋯N4^iii^	0.91 (2)	2.01 (2)	2.916 (2)	172 (1)

Symmetry codes: (i) 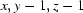; (ii) 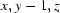; (iii) 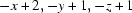.

**Fig. 1.**
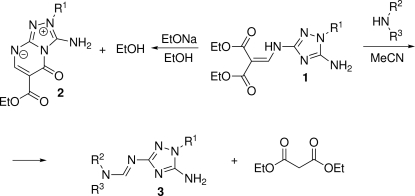
Reactions of the compounds 1 with sodium ethoxide and aliphatic amines.

**Fig. 2.**
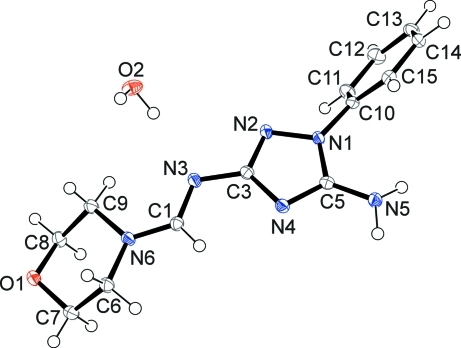
The molecular structure of the title compound with the atom numbering scheme. Displacement ellipsoids are drawn at the 50% probability level.

**Fig. 3.**
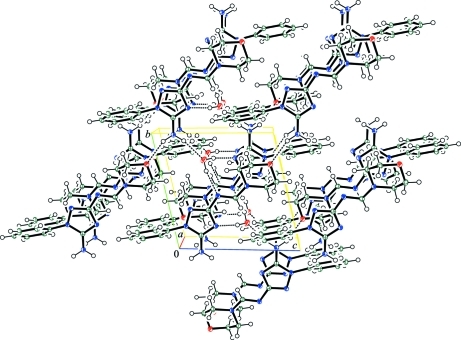
Molecular packing in the crystal, viewed along the a axis. Hydrogen bonds are shown as dashed lines.

**Fig. 4.**
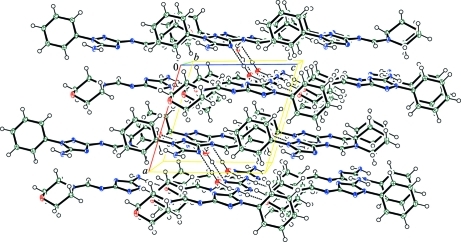
The crystal packing of the title compound viewed approximately along the b axis and showing double layers parallel to the bc planes. Hydrogen bonds are shown as dashed lines.

## supplementary crystallographic information

### Comment

Recently, we have reported a simple method for the synthesis of mesoionic 3-amino-5-oxo-2-*R*-2,5-dihydro-[1,2,4]triazolo[4,3-*a*]pyrimidines by heating of *N*-(5-amino-1-*R*-1,2,4-triazol-3-yl)-substituted enaminoesters in alkaline alcoholic solutions (Chernyshev *et al.*, 2010). In the analogous conditions, *N*-(5-amino-1-*R*-1,2,4-triazol-3-yl)-substituted aminomethylene malonates 1 (Fig. 1) furnished mesoionic 3-amino-6-(ethoxycarbonyl)-2-*R*-5-oxo-5*H*-[1,2,4]triazolo[4,3- *a*]pyrimidines 2 in high yield (Astakhov & Chernyshev, 2010). However, when the compounds 1 were heated with aliphatic amines in acetonitrile, nucleophilic substitution of malonic ester affording the amidines 3 was observed instead of the expected reactions of heterocyclization or amidation (Fig. 1). This reaction is analogous to the previously described intramolecular heterocyclizations of N-substituted aminomethylene malonates (Sunder & Peet, 1980;Yamazaki *et al.*, 1988; Selic *et al.*, 1998, 2000; Tkachev *et al.*, 2007). However, we could find the intermolecular variant of the reaction in two publications (Rajappa *et al.*, 1970; Bao *et al.*, 2008), only. Good yields of the compounds 3 allow to expect that the reaction will be a useful tool for the selective synthesis of *N*-hetaryl substituted formamidines. Analogous compounds are valuable building blocks for the preparation of annulated heterocycles (Dolzhenko *et al.*, 2007, 2008**a*,b*).

For unambiguous confirmation of structure of the compounds 3 (Fig. 1), we performed an X-ray investigation of the title compound. In accordance with the X-ray diffraction data (Fig. 2), the benzene and triazole rings are not coplanar, the dihedral angle is 54.80 (5)°. Bond lengths and angles in the triazole cycle are within the normal ranges and are comparable with those found in the other substituted 3,5-diamino-1,2,4-triazoles (Ried *et al.*, 1983; Dunstan *et al.*, 1998; Chernyshev *et al.*, 2006, 2007, 2009). The nitrogen atom of the amino group is in a trigonal pyramidal configuration (sum of valence angles is 349.8°) and deviates from the triazole plane by only 0.020 (2) Å. Conjugation between the unshared electron pair of N5 and the π system of the triazole fragment leads to a shortening of the N5—C5 bond (1.352 (2) Å) relative to the standard length of a purely single Nsp2-Csp2 bond (1.43–1.45 Å) (Burke-Laing & Laing,1976; Allen *et al.*, 1987). The N3 atom deviates from the least-squares plane of the triazole cycle by 0.056 (2) Å. The dihedral angle between the planes of the triazole cycle and amidine fragment (H1/C1/N3/N6) of the molecule is 8.66 (7)°. The amidine fragment is in the *E* configuration, as in the majority of other (het)aryl substituted formamidines (Cambridge Structural Database, Version 5.31 of November 2009, including updates up to August 2010, Allen, 2002). Although the formally single bond N6—C1 (1.337 (2) Å) is longer than the double bond N3—C1 (1.297 (2) Å), it is sufficiently shorter than the purely single Nsp2-Csp2 bond (1.43–1.45 Å) (Burke-Laing & Laing,1976; Allen *et al.*, 1987). Apparently, that is caused by conjugation of the N6 atom lone pair with the N3—C1 double bond, analogously to the other hetaryl substituted formamidines (Ryng & Glowiak, 1998; Kurbatov *et al.*, 2006; Xie *et al.*, 2007; Lyakhov *et al.*, 2008; Quiroga *et al.*, 2010). Atom N6 of morpholine cycle has a slightly pyramidalized trigonal configuration (sum of valence angles is 359.1°). The morpholine ring adopts the usual chair conformation.

In the crystal, the molecules C_13_H_16_N_6_O with the parallel oriented triazole and morpholine cycles form stacks along the *a* axis of the triclinic cell (Fig. 3). The nearest molecules in the stacks adopt inverse orientation, i. e. they are space related by the inversion centres with coordinates [0, 0, 0]. The pairs of the nearest inversely oriented molecules in the stacks are connected with two water molecules located between them by means of the hydrogen bonds O2—H2A···N3 and O2—H2B···N4 (Table 1). These stacks together with the water molecules form rows which are parallel to the (011) plane (Fig. 3). In these rows the inversely oriented molecules C_13_H_16_N_6_O of the neighboring stacks are linked with each other by the chains of N5—H5A···O1 hydrogen bonds. The rows are connected with one another by the system of N5—H5B···O2 hydrogen bonds (Table 1). In the crystal, parallel to (100), one can see two types of molecular layers consisting of the molecules C_13_H_16_N_6_O (Fig. 4). The adjacent layers are related by the inversion centres. In the each layer the nearest molecules C_13_H_16_N_6_O are displaced from each other by the cell parameter along the *b* and *c* axes. The neighbouring layers from both sides of the (100) crystallographic planes are pairwise linked by the O2—H2A···N3, O2—H2B···N4 and N5—H5B···O2 hydrogen bonds. Thus, the crystal structure consists of the C_13_H_16_N_6_O×H_2_O molecular double layers in the direction of normal to the (100) plane.

### Experimental

The crystals of *N*^3^-[(*E*)-morpholin-4-ylmethylidene]-1-phenyl-1*H*-1,2,4- triazole-3,5-diamine hydrate suitable for X-ray analysis were grown by slow evaporation from 1:9 water: acetonitrile mixture at room temperature. The title compound was prepared by the following procedure.

A mixture of diethyl 2-(((5-amino-1-phenyl-1*H*-1,2,4-triazol-3-yl)amino)methylene)malonate (1a, *R*^1^ = Ph, 0.69 g, 2 mmol), morpholine (0.37 g, 4.2 mmol) and acetonitrile (5 ml) was refluxed for 5 h, then cooled to 0 °C. The precipitate formed was isolated by filtration, recrystallized from acetonitrile and dried at 130 °C to give 0.46 g (84% yield) of white powder, m. p. 208–208.5 °C. Spectrum ^1^H NMR (300 MHz), *δ*: 3.42–3.61 (m, 8H, 4CH_2_), 6.21 (s, 2H, NH_2_), 7.25–7.53 (m, 5H, Ph), 8.26 (s, 1H, CH). Spectrum ^13^C NMR (125 MHz), *δ*: 42.52, 48.58, 65.44, 66.57, 121.68, 125.75, 129.16, 137.71, 153.59 (C5 of triazole), 155.03 (N—CH═N), 163.52 (C3 of triazole). MS (EI, 70 eV), *m*/*z* (%): 272 (*M*^+^, 100), 241 (25), 186 (17), 175 (17), 77 (27). Anal. Calcd for C_13_H_16_N_6_O: C 57.34; H 5.92; N 30.86. Found: C 57.35; H 5.94; N 30.88.

For the preparation of compound 1a a solution of 3,5-diamino-1-phenyl-1,2,4-triazole (1.05 g, 6 mmol) and diethyl 2-(ethoxymethylene)malonate (1.56 g, 7.2 mmol) in EtOH (5 ml) was refluxed for 2 h, then water (5 ml) was added. After cooling to 20 °C, the precipitate formed was isolated by filtration and recrystallized from ethanol. Yield 2.07 g (97%) of white powder, m. p. 140–141 °C. Spectrum ^1^H NMR (300 MHz) *δ*: 1.22 (t, *J* = 6.9 Hz, 3H, OCH_2_CH_3_), 1.24 (t, *J* = 6.9 Hz, 3H, OCH_2_CH_3_), 4.12 (q, *J* = 6.9 Hz, 2H, OCH_2_CH_3_), 4.20 (q, *J* = 6.9 Hz, 2H, OCH_2_CH_3_), 6.84 (s, 2H, NH_2_), 7.33–7.54 (m, 5H, Ph), 8.53 (d, *J*=13.4 Hz, 1H, CH), 10.56 (d, *J*=13.4 Hz, 1H, NH). MS (EI, 70 eV), *m*/*z* (%): 345 (*M*^+^, 21), 254 (18), 253 (99), 186 (21), 119 (37), 105 (16), 91 (34), 77 (100). Anal. Calcd for C_16_H_19_N_5_O_4_: C, 55.64; H, 5.55; N, 20.28. Found: C, 55.81; H, 5.62; N, 20.04. Starting 3,5-diamino-1-phenyl-1,2,4-triazole was synthesized by known method (Steck, *et al.*, 1958).

### Refinement

The hydrogen atoms of NH_2_ group and H_2_O molecule were found in difference Fourier synthesis and were refined in isotropic approximation. C-bound H atoms were geometrically positioned (C—H 0.93-0.97 Å) and refined in riding model approximation, with *U*_iso_(H) = 1.2 *U*_eq_(C).

### Crystal data

**Table:**

C_13_H_16_N_6_O·H_2_O	*Z* = 2
*M**_r_* = 290.33	*F*(000) = 308
Triclinic, *P*1	*D*_x_ = 1.377 Mg m^−^^3^
Hall symbol: -P 1	Melting point: 208 K
*a* = 8.7886 (7) Å	Mo *K*α radiation, λ = 0.71073 Å
*b* = 9.0100 (7) Å	Cell parameters from 334 reflections
*c* = 9.4373 (7) Å	θ = 3–26°
α = 99.938 (1)°	µ = 0.10 mm^−^^1^
β = 105.933 (1)°	*T* = 100 K
γ = 95.331 (1)°	Plate, colourless
*V* = 700.00 (9) Å^3^	0.55 × 0.30 × 0.25 mm

### Data collection

**Table:**

Bruker APEXII CCD area-detector diffractometer	2724 independent reflections
Radiation source: fine-focus sealed tube	2510 reflections with *I* > 2σ(*I*)
graphite	*R*_int_ = 0.015
ω scans	θ_max_ = 26.0°, θ_min_ = 2.3°
Absorption correction: multi-scan (*SADABS*; Bruker, 2004)	*h* = −10→10
*T*_min_ = 0.948, *T*_max_ = 0.976	*k* = −11→11
5231 measured reflections	*l* = −11→11

### Refinement

**Table:**

Refinement on *F*^2^	Primary atom site location: structure-invariant direct methods
Least-squares matrix: full	Secondary atom site location: difference Fourier map
*R*[*F*^2^ > 2σ(*F*^2^)] = 0.034	Hydrogen site location: difference Fourier map
*wR*(*F*^2^) = 0.088	H atoms treated by a mixture of independent and constrained refinement
*S* = 1.00	*w* = 1/[σ^2^(*F*_o_^2^) + (0.0447*P*)^2^ + 0.3407*P*] where *P* = (*F*_o_^2^ + 2*F*_c_^2^)/3
2724 reflections	(Δ/σ)_max_ < 0.001
206 parameters	Δρ_max_ = 0.18 e Å^−^^3^
0 restraints	Δρ_min_ = −0.28 e Å^−^^3^

### Special details

**Table:**

Geometry. All e.s.d.'s (except the e.s.d. in the dihedral angle between two l.s. planes) are estimated using the full covariance matrix. The cell e.s.d.'s are taken into account individually in the estimation of e.s.d.'s in distances, angles and torsion angles; correlations between e.s.d.'s in cell parameters are only used when they are defined by crystal symmetry. An approximate (isotropic) treatment of cell e.s.d.'s is used for estimating e.s.d.'s involving l.s. planes.
Refinement. Refinement of *F*^2^ against ALL reflections. The weighted *R*-factor *wR* and goodness of fit *S* are based on *F*^2^, conventional *R*-factors *R* are based on *F*, with *F* set to zero for negative *F*^2^. The threshold expression of *F*^2^ > σ(*F*^2^) is used only for calculating *R*-factors(gt) *etc*. and is not relevant to the choice of reflections for refinement. *R*-factors based on *F*^2^ are statistically about twice as large as those based on *F*, and *R*- factors based on ALL data will be even larger.

### Fractional atomic coordinates and isotropic or equivalent isotropic displacement parameters (Å^2^)

**Table:**

	*x*	*y*	*z*	*U*_iso_*/*U*_eq_	
O1	0.71171 (11)	0.75346 (9)	0.92066 (9)	0.0197 (2)	
N1	0.75642 (11)	0.22365 (11)	0.11933 (11)	0.0140 (2)	
N2	0.73939 (12)	0.36780 (11)	0.19018 (11)	0.0149 (2)	
N4	0.82835 (11)	0.22141 (11)	0.36142 (11)	0.0140 (2)	
N5	0.83821 (12)	−0.00456 (11)	0.19122 (12)	0.0163 (2)	
H5A	0.7927 (19)	−0.0581 (18)	0.0975 (19)	0.025 (4)*	
H5B	0.8474 (19)	−0.0575 (18)	0.2638 (19)	0.027 (4)*	
N3	0.77750 (11)	0.48235 (11)	0.44096 (11)	0.0146 (2)	
N6	0.79432 (12)	0.56639 (11)	0.69189 (11)	0.0154 (2)	
C5	0.80778 (13)	0.13965 (13)	0.22417 (12)	0.0132 (2)	
C3	0.78422 (13)	0.35886 (13)	0.33343 (13)	0.0131 (2)	
C1	0.81153 (13)	0.46393 (13)	0.57891 (13)	0.0139 (2)	
H1	0.8498	0.3750	0.6003	0.017*	
C6	0.85744 (15)	0.55214 (13)	0.84809 (13)	0.0179 (3)	
H6A	0.9620	0.6136	0.8926	0.021*	
H6B	0.8697	0.4468	0.8515	0.021*	
C7	0.74577 (17)	0.60386 (14)	0.93772 (14)	0.0225 (3)	
H7A	0.6468	0.5329	0.9031	0.027*	
H7B	0.7949	0.6046	1.0433	0.027*	
C8	0.63106 (14)	0.74989 (14)	0.76612 (13)	0.0173 (2)	
H8A	0.5986	0.8484	0.7557	0.021*	
H8B	0.5356	0.6743	0.7322	0.021*	
C9	0.73945 (15)	0.71137 (13)	0.66976 (13)	0.0179 (3)	
H9A	0.6819	0.7038	0.5646	0.021*	
H9B	0.8306	0.7912	0.6975	0.021*	
C10	0.71449 (14)	0.18521 (13)	−0.04071 (12)	0.0141 (2)	
C11	0.56426 (14)	0.20692 (14)	−0.12420 (13)	0.0179 (3)	
H11	0.4903	0.2391	−0.0761	0.021*	
C12	0.52581 (15)	0.18009 (14)	−0.27987 (14)	0.0206 (3)	
H12	0.4252	0.1937	−0.3365	0.025*	
C13	0.63660 (15)	0.13300 (14)	−0.35193 (13)	0.0188 (3)	
H13	0.6114	0.1180	−0.4562	0.023*	
C14	0.78489 (14)	0.10853 (13)	−0.26786 (13)	0.0167 (2)	
H14	0.8581	0.0749	−0.3162	0.020*	
C15	0.82470 (14)	0.13401 (13)	−0.11178 (13)	0.0154 (2)	
H15	0.9239	0.1170	−0.0555	0.019*	
O2	0.88944 (11)	0.77890 (11)	0.38883 (10)	0.0217 (2)	
H2A	0.840 (2)	0.688 (2)	0.389 (2)	0.041 (5)*	
H2B	0.979 (2)	0.788 (2)	0.467 (2)	0.048 (5)*	

### Atomic displacement parameters (Å^2^)

**Table:**

	*U*^11^	*U*^22^	*U*^33^	*U*^12^	*U*^13^	*U*^23^
O1	0.0301 (5)	0.0169 (4)	0.0141 (4)	0.0069 (4)	0.0092 (4)	0.0025 (3)
N1	0.0184 (5)	0.0133 (5)	0.0105 (5)	0.0043 (4)	0.0048 (4)	0.0016 (4)
N2	0.0192 (5)	0.0134 (5)	0.0131 (5)	0.0043 (4)	0.0063 (4)	0.0015 (4)
N4	0.0147 (5)	0.0149 (5)	0.0122 (5)	0.0028 (4)	0.0039 (4)	0.0027 (4)
N5	0.0218 (5)	0.0148 (5)	0.0120 (5)	0.0049 (4)	0.0037 (4)	0.0027 (4)
N3	0.0166 (5)	0.0150 (5)	0.0128 (5)	0.0035 (4)	0.0055 (4)	0.0018 (4)
N6	0.0205 (5)	0.0156 (5)	0.0114 (5)	0.0062 (4)	0.0052 (4)	0.0034 (4)
C5	0.0108 (5)	0.0158 (5)	0.0132 (5)	0.0012 (4)	0.0037 (4)	0.0036 (4)
C3	0.0117 (5)	0.0147 (5)	0.0135 (5)	0.0018 (4)	0.0047 (4)	0.0029 (4)
C1	0.0140 (5)	0.0134 (5)	0.0147 (5)	0.0023 (4)	0.0047 (4)	0.0025 (4)
C6	0.0241 (6)	0.0168 (6)	0.0121 (6)	0.0059 (5)	0.0032 (5)	0.0037 (4)
C7	0.0373 (7)	0.0187 (6)	0.0164 (6)	0.0082 (5)	0.0133 (5)	0.0061 (5)
C8	0.0177 (6)	0.0177 (6)	0.0163 (6)	0.0041 (4)	0.0054 (5)	0.0021 (4)
C9	0.0252 (6)	0.0171 (6)	0.0146 (6)	0.0087 (5)	0.0082 (5)	0.0053 (4)
C10	0.0181 (6)	0.0128 (5)	0.0112 (5)	0.0011 (4)	0.0044 (4)	0.0029 (4)
C11	0.0161 (6)	0.0215 (6)	0.0172 (6)	0.0039 (5)	0.0064 (5)	0.0039 (5)
C12	0.0176 (6)	0.0266 (6)	0.0160 (6)	0.0031 (5)	0.0014 (5)	0.0060 (5)
C13	0.0243 (6)	0.0190 (6)	0.0113 (5)	−0.0017 (5)	0.0043 (5)	0.0025 (4)
C14	0.0211 (6)	0.0143 (5)	0.0164 (6)	0.0012 (4)	0.0099 (5)	0.0016 (4)
C15	0.0161 (5)	0.0136 (5)	0.0168 (6)	0.0024 (4)	0.0047 (4)	0.0036 (4)
O2	0.0204 (5)	0.0227 (5)	0.0224 (5)	0.0028 (4)	0.0027 (4)	0.0121 (4)

### Geometric parameters (Å, °)

**Table:**

O1—C8	1.4292 (14)	C7—H7A	0.9700
O1—C7	1.4338 (15)	C7—H7B	0.9700
N1—C5	1.3536 (15)	C8—C9	1.5109 (16)
N1—N2	1.3956 (13)	C8—H8A	0.9700
N1—C10	1.4238 (14)	C8—H8B	0.9700
N2—C3	1.3193 (15)	C9—H9A	0.9700
N4—C5	1.3305 (15)	C9—H9B	0.9700
N4—C3	1.3783 (15)	C10—C11	1.3900 (16)
N5—C5	1.3517 (15)	C10—C15	1.3906 (16)
N5—H5A	0.893 (17)	C11—C12	1.3858 (17)
N5—H5B	0.890 (17)	C11—H11	0.9300
N3—C1	1.2968 (15)	C12—C13	1.3897 (18)
N3—C3	1.3880 (15)	C12—H12	0.9300
N6—C1	1.3367 (15)	C13—C14	1.3866 (17)
N6—C6	1.4584 (14)	C13—H13	0.9300
N6—C9	1.4623 (15)	C14—C15	1.3893 (16)
C1—H1	0.9300	C14—H14	0.9300
C6—C7	1.5167 (17)	C15—H15	0.9300
C6—H6A	0.9700	O2—H2A	0.89 (2)
C6—H6B	0.9700	O2—H2B	0.91 (2)
			
C8—O1—C7	109.34 (9)	H7A—C7—H7B	108.1
C5—N1—N2	109.50 (9)	O1—C8—C9	110.45 (9)
C5—N1—C10	130.86 (10)	O1—C8—H8A	109.6
N2—N1—C10	119.58 (9)	C9—C8—H8A	109.6
C3—N2—N1	101.95 (9)	O1—C8—H8B	109.6
C5—N4—C3	103.04 (9)	C9—C8—H8B	109.6
C5—N5—H5A	117.9 (10)	H8A—C8—H8B	108.1
C5—N5—H5B	116.4 (10)	N6—C9—C8	109.22 (9)
H5A—N5—H5B	115.6 (14)	N6—C9—H9A	109.8
C1—N3—C3	116.52 (10)	C8—C9—H9A	109.8
C1—N6—C6	121.12 (10)	N6—C9—H9B	109.8
C1—N6—C9	122.19 (10)	C8—C9—H9B	109.8
C6—N6—C9	115.74 (9)	H9A—C9—H9B	108.3
N4—C5—N5	126.03 (10)	C11—C10—C15	120.75 (10)
N4—C5—N1	110.18 (10)	C11—C10—N1	118.68 (10)
N5—C5—N1	123.76 (10)	C15—C10—N1	120.49 (10)
N2—C3—N4	115.33 (10)	C12—C11—C10	119.35 (11)
N2—C3—N3	118.89 (10)	C12—C11—H11	120.3
N4—C3—N3	125.73 (10)	C10—C11—H11	120.3
N3—C1—N6	123.28 (11)	C11—C12—C13	120.41 (11)
N3—C1—H1	118.4	C11—C12—H12	119.8
N6—C1—H1	118.4	C13—C12—H12	119.8
N6—C6—C7	110.52 (10)	C14—C13—C12	119.80 (11)
N6—C6—H6A	109.5	C14—C13—H13	120.1
C7—C6—H6A	109.5	C12—C13—H13	120.1
N6—C6—H6B	109.5	C13—C14—C15	120.40 (11)
C7—C6—H6B	109.5	C13—C14—H14	119.8
H6A—C6—H6B	108.1	C15—C14—H14	119.8
O1—C7—C6	110.56 (10)	C14—C15—C10	119.25 (11)
O1—C7—H7A	109.5	C14—C15—H15	120.4
C6—C7—H7A	109.5	C10—C15—H15	120.4
O1—C7—H7B	109.5	H2A—O2—H2B	101.2 (16)
C6—C7—H7B	109.5		
			
C5—N1—N2—C3	0.70 (11)	C8—O1—C7—C6	62.51 (13)
C10—N1—N2—C3	178.22 (9)	N6—C6—C7—O1	−52.83 (13)
C3—N4—C5—N5	178.66 (11)	C7—O1—C8—C9	−64.75 (12)
C3—N4—C5—N1	0.87 (12)	C1—N6—C9—C8	142.14 (11)
N2—N1—C5—N4	−1.03 (12)	C6—N6—C9—C8	−48.90 (13)
C10—N1—C5—N4	−178.18 (10)	O1—C8—C9—N6	56.40 (13)
N2—N1—C5—N5	−178.89 (10)	C5—N1—C10—C11	124.87 (13)
C10—N1—C5—N5	3.96 (19)	N2—N1—C10—C11	−52.04 (14)
N1—N2—C3—N4	−0.16 (12)	C5—N1—C10—C15	−58.21 (16)
N1—N2—C3—N3	−177.65 (9)	N2—N1—C10—C15	124.87 (11)
C5—N4—C3—N2	−0.43 (12)	C15—C10—C11—C12	−1.36 (18)
C5—N4—C3—N3	176.86 (10)	N1—C10—C11—C12	175.55 (10)
C1—N3—C3—N2	175.10 (10)	C10—C11—C12—C13	−0.46 (18)
C1—N3—C3—N4	−2.11 (16)	C11—C12—C13—C14	1.84 (18)
C3—N3—C1—N6	−172.89 (10)	C12—C13—C14—C15	−1.42 (18)
C6—N6—C1—N3	−170.20 (11)	C13—C14—C15—C10	−0.37 (17)
C9—N6—C1—N3	−1.83 (17)	C11—C10—C15—C14	1.77 (17)
C1—N6—C6—C7	−143.44 (11)	N1—C10—C15—C14	−175.08 (10)
C9—N6—C6—C7	47.47 (14)		

### Hydrogen-bond geometry (Å, °)

**Table:**

*D*—H···*A*	*D*—H	H···*A*	*D*···*A*	*D*—H···*A*
N5—H5A···O1^i^	0.89 (2)	2.08 (2)	2.929 (2)	159 (1)
N5—H5B···O2^ii^	0.89 (2)	2.04 (2)	2.906 (2)	164 (1)
O2—H2A···N3	0.89 (2)	2.07 (2)	2.929 (2)	164 (1)
O2—H2B···N4^iii^	0.91 (2)	2.01 (2)	2.916 (2)	172 (1)

Symmetry codes: (i) *x*, *y*−1, *z*−1; (ii) *x*, *y*−1, *z*;  (iii) −*x*+2, −*y*+1, −*z*+1.
